# Using IoT Assistive Technologies for Older People Non-Invasive Monitoring and Living Support in Their Homes

**DOI:** 10.3390/ijerph19105890

**Published:** 2022-05-12

**Authors:** Sorin-Aurel Moraru, Adrian Alexandru Moșoi, Dominic Mircea Kristaly, Ionuț Moraru, Vlad Ștefan Petre, Delia Elisabeta Ungureanu, Liviu Marian Perniu, Dan Rosenberg, Maria Elena Cocuz

**Affiliations:** 1 Department of Automatics and Information Technology, Transilvania University of Brașov, B-dul Eroilor 29, 500036 Brașov, Romania; vlad.petre@unitbv.ro (V.Ș.P.); deliau@unitbv.ro (D.E.U.); liviu.perniu@unitbv.ro (L.M.P.); dan.rosenberg@unitbv.ro (D.R.); 2 Department of Psychology, Education and Teacher Training, Transilvania University of Brașov, B-dul Eroilor 29, 500036 Brașov, Romania; adrian.mosoi@unitbv.ro; 3 Department of Informatics, King’s College London, 30 Aldwych, London WC2B 4BG, UK; imoraru@lincoln.ac.uk; 4 School of Computer Science, University of Lincoln, Brayford Pool Campus, Lincoln LN6 7TS, UK; 5 Department of Fundamental Disciplines and Clinical Prevention, Transilvania University of Brașov, B-dul Eroilor 29, 500036 Brașov, Romania; maria.cocuz@unitbv.ro

**Keywords:** AAL programme, REST, sensors, microservices, behavioural analysis models, ABAS, cloud, Agile

## Abstract

Many western societies are confronted with issues in planning and adapting their health policies due to an ageing population living alone. The “NOt Alone at Home—NOAH” project aimed to involve older people in the Agile co-creation of services for a collaborative monitoring and awareness notification for remote caregivers. Our research aim was to create a scalable and modern information system that permitted a non-invasive monitorization of the users for keeping their caregivers up to date. This was done via a cloud IoT (Internet of Things), which collects and processes data from its domotic sensors. The notifications generated by the system, via the three applications we developed (NOAH/NOAH Care/Admin Centre), offer caregivers an easy way of detecting changes in the day-to-day behaviour and activities of their patients, giving them time to intervene in case of abnormal activity. Such an approach would lead to a longer and more independent life for the older people. We evaluated our system by conducting a year-long pilot-study, offering caregivers constant information from the end-users while still living independently. For creating our pilot groups, we used the ABAS (Adaptive Behaviour Assessment System) II, which we then matched with the pre-profiled Behavioral Analysis Models of older people familiar with modern communication devices. Our results showed a low association between daily skills and the sensors we used, in contrast with the results from previous studies done in this field. Another result was efficiently capturing the behaviour changes that took place due to the COVID-19 Lockdown measures.

## 1. Introduction

An ageing population is a vital aspect in planning and adapting public health policies within a society. The projects developed under the European Active Assisted Living (AAL) program aim to keep people connected, healthy and active until old age, under the slogan: “Ageing Well in the Digital World”, providing older people with a safe, yet independent living environment. The most important aspect within this framework is the activities of daily living (ADL) component, a component with a significant impact on the adaptation of the environment to the older people’s needs, taking into account also the psycho-social factors through human–technology interaction.

The research presented in the following section was motivated by the need to extend the time that older people spent actively living in their home, thereby reducing institutionalization. The Cloud/Edge modern technologies bring artificial intelligence and centralized resource-intensive computation in cooperation with distributed IoT (Internet of Things) WSAN (Wireless Sensors and Actuators Networks), which have become pervasive in smart buildings. The older-people-centered approach was supported not only by the Agile co-creation of assistive services, but also by the psychological assessment of volunteers based on the ABAS (Adaptive Behaviour Assessment System) methods of selection for the pilot groups and on Behavioral Analysis Models pre-profiling (BAM “tuning”).

The Active and Assisted Living (AAL) program supports projects through which applied research on innovative ICT-based services is carried out and implemented for the benefits of aging well, targeted to older people, who are increasing in number and in the fully deserved esteem of modern society.

The AAL program financed the “NOt Alone at Home—NOAH” project, which contributes with solutions for older people’s independent living, providing them the opportunity to manage their daily activities self-sufficiently. This can be done by ensuring a permanent connection of the end-users with caregivers who can help them (relatives, friends, professional caregivers) and by providing helpful tools to keep them safe and by providing them with increased confidence.

Being able to live independently also depends on subjective perceptions, such as feeling safe and comfortable while alone at home. For instance, in the AAL Strategic Research Agenda from March 2010 [1], a holistic list of requisites for independent life, was given, which includes aspects such as:

- a secure environment;
- constant contact with friends and family (to provide reassurance);
- physical, social and mental stimulation;
- knowledge that, if needed, carers will come to support them;
- relying on appropriate responses when in trouble.

We tried to address all these needs in the NOAH project, by developing a socio-technological system and embedding a practical, usable and accessible monitoring technology, which is not intrusive and is suitable for inspiring users’ trust.

This was achieved by the implementation of the users’ suggestions and feedback received during co-design sessions.

Our research aim was to create a scalable and modern information system that permitted a non-invasive monitorization of the users for keeping their caregivers up to date. This was done via a cloud IoT, which collected and processed data from its domotic sensors. The notifications generated by the system needed to offer caregivers an easy way of detecting changes in the day-to-day behaviour and activities of their patients. This would offer them ample time to intervene in case of abnormal activity, which would lead to a longer and more independent life for the older people.

The rest of the paper is organized as follows. In Section 2, we present the background and related works of our project within the AAL context and, based on these, the hypotheses to be proved by our work. Section 3 is dedicated to the NOAH solution, with the innovative platform, the unified Cloudant database and Kibana, as an advanced analytical tool. Section 4 shows the system architecture, prototyping and the microservices-based variant. In Section 5, the database is described, with the tables storing the data being detailed. Section 6 presents the sensors comprising the NOAH kit and its associated library and communication. Two mobile applications for the end-users and caregivers (NOAH and NOAHCare) and one web application for administrators (Admin Centre) are presented in Section 7. Section 8 contains a detailed explanation about the methods, instruments, procedure and data analysis used in the project. Section 9 evaluates the descriptive and correlation results and analyzes the differences between the periods. Section 10 is dedicated to the discussion and some limitations, and finally, in Section 11, the work finishes with some conclusions.

## 2. Background and Related Works

The AAL classification comprises four basic categories: smart homes, smart nurses, portable devices and robotics [2], integrated in different kinds of solutions enabled by technologies in areas such as health and care, living and building and safety and security.

All these categories have warnings about data security laws and common frameworks for interoperability, privacy, security management [3] and about confidentiality with information security and unauthorized threats to accessibility [4]. Data collection is based on the critical performance of Internet-aware technologies for monitoring older people, such as the city/home data capture layer, the centralized Cloud-based data, the management repository and the risk analysis and prediction module [5].

The Internet of Things (IoT) aims to integrate various sensors and applications that allow users to share information, data and resources and which can provide information about a person’s level of functioning, by measuring and analyzing physiological and environmental parameters. These IoT systems are intended for use in areas such as health care, hospital health systems and safety and sound monitoring [6].

Such systems would trigger alarms in the case of some medical conditions that are critical for the user. For example, if the patient does not respond to a prescribed medication, the Cloud interprets data collected by different sensors and triggers an alarm, so the medical staff can intervene [7].

This type of architecture and use of that Cloud environment is a technology that has become more and more popular among researchers due to its great flexibility and power [8,9].

The context of IoT-based systems oriented to the monitoring of older people has been exploited in several works following a similar design and combining both hardware and software components of many forms, being implemented with different technologies. Many of these papers follow a similar pattern, consisting of a cloud-based architecture that involves sensors of any kind that collect data into the cloud to be processed and provided to the users involved.

In recent years, several research projects started using IoT-based systems that aim to monitor older people. These works follow a similar design and combine both hardware and software components but use a wide variety of technologies. Many of these papers follow a similar framework, consisting of a cloud-based architecture that involves sensors of any kind that collect data into the cloud. This data is then processed and provided to the users.

CoSHIE [10], for example, is a system presented that gathers data from non-invasive sensors through a home gateway into the cloud, where they are processed and stored in a non-relational database. However, it has some limitations regarding the direct monitoring of older people by their caregivers. Similarly, SW-SHMS [11] is a system separated into three main components: a user environment collecting data from wearable sensors through a gateway and a cloud datacenter to compute the data and provide information as alerts to the third component, which is a monitoring platform for the caregivers. The City4Age Project [12] is intended to support older people’s daily life but on a different scale, being oriented also to outdoor monitoring. It is built on a similar architecture, which was enhanced by adding a behavioral analysis and risk management component. Additionally, Wang, Y. and Jang, S. [13] presented another way of implementing such a system and described it as being composed of a home gateway (RaspberryPi 3B+) directly connected with sensors (via Arduino UNO). The gateway communicates with a cloud component to save the data into a non-relational database and provide information to its users through mobile or web applications.

These projects mainly have the same goal, and even though they follow similar architectures, they combine different technologies and present some particularities for different topics. Nevertheless, in terms of development, these systems have been divided into more independent components, which can be implemented separately and incrementally, presenting a standard communication interface for interaction among them.

An important goal is the independent operation of these systems, reducing the strain on doctors (or specialized medical staff) by using biometric information obtained from users. This information could be used as a supplement to existing data acquired by relatives/caregivers in order to obtain a better image that enables comprehensive care for older people [14,15]. A possible integration of these systems could be reached using an interdisciplinary approach, by standardizing the data used on human-oriented studies. Two key directions in the implementation of IoT systems would be a) digitization of public health systems and enhanced security; social isolation prevention; improving quality of life and b) technology-based economic development for older people; development of new markets for smart devices; encouragement of private investment in the smart IoT [15].

All these references provide valuable information about the guidelines of the AAL program from the perspective of the use of sensors and IT equipment but less information about the involvement of older people in activities that require physical activities to maintain executive functions. Small positive effects were observed, regardless of health and physical activity, for the older people who were initially sedentary; they seemed to benefit from training interventions of exercises in relation to the executive function performed [16]. The effects of a sedentary lifestyle were observed, especially during the COVID-19 crisis, and one of the recommendations, especially important for the older population, was to maintain regular physical activity during self-isolation to prevent frailty and deterioration of health [17]. Together with these aspects of systems’ interconnection and the interdisciplinary approach, the discussion remains open about the lifetime of using these methods and their benefits in long-term studies exploring effects and benefits [4].

The development and implementation of life improvement systems through AAL programs is an ongoing challenge. New data was continuously collected during and after the completion of the project [18]. In this regard, Calvaresi, D. et al. [19] indicated two clear needs in streamlining these systems by understanding and validating new solutions and re-evaluating the relationship between the system user and the proposed solutions.

Sensors are fixed in the same position on household objects (bed, refrigerator, living room, bathroom and bed) being able to detect human activity in the home. The sensors can analyze the activity levels of the older people in order to identify behavioral changes that can detect abnormal situations [20,21].

Co-creation allows the involvement of users [22] in a project team by using personal experience to design solutions using the ideas of future users in possible real solutions [23] which will contribute to user well-being [24]. In carrying out a co-design, the involved researchers must know the objectives of the project in order to match the development with them [25]. In the NOAH project, the participatory validation of ideas [26] was accomplished based on information generated in the context of “health and sustainability” for people lives. These users were encouraged to actively participate in the development of the future products [27]. The co-creation was carried out in successful AAL projects, such as “CaMeli” [28], “Rose” [29] and “Cognivitra” [30], by validating the results with the help of all users involved (end-users, caregivers and stakeholders).

The social evaluation and technical approach will be considered together for providing more coherence and displaying the interconnected nature of our methodology.

After reviewing the studies conducted on the topic mentioned above, with results presented in [19,20], we decided on the following research questions:

(1) Do domotic sensors have long-term relevance and utility?
(2) Did using the system lead to changes in the lifestyle of the older people participating in our pilot?
(3) Was the users’ behaviour, in their homes during their daily live, affected by the Covid crisis?

## 3. Solution Overview

Our research was focused on improving elder people’s independence and providing relief for caregivers based on technological designing and solution implementing.

The innovative platform developed within the project was implemented as an integrated solution for the use of mobile devices in a Cloud computing environment. This environment allows the import and distribution of developed applications, allowing users to access them directly.

The platform:

- Uses open-source software products, both in terms of programming languages, programming environments, tools, databases and architectures offered and as standardization, to ensure interoperability and interchange;
- Ensures the safe storage of data, the scalability of resources necessary for the correct and uninterrupted operation of applications and the possibility of using advanced data analysis services to obtain the necessary information, both from the analysis of stored data and from the devices from which they are collected;
- Offers the possibility to choose the desired software products and to optimize the maximum costs by the fact that only exactly what is used is paid (the Pay Per Use, PPU, principle).

The solution optimizes five important criteria: benefits, costs, flexibility, scalability and risks. It integrates services; proves usability; uses state-of-the-art devices and technologies (Section 4) and collects, stores and processes data (Section 5, Section 6 and Section 7), ensuring the security of the system regarding the use of devices.

According to its architecture, the NOAH system implements the client–server model, combining multiple technologies to achieve the best outcome. The core purpose of monitoring implies that there are sensors gathering data and feeding them to a server, where they are processed in several ways and, in the end, delivered to a final user (via mobile applications) to be interpreted [31,32]. For these features, the NOAH system takes advantages of Cloud technology, meaning that data collection, processing and delivery are achieved in the Cloud [11].

A unified database was designed, developed, and implemented using the IBM Cloud with Cloudant database. Basic algorithms were implemented for the behavioral analysis module (BAM) and the Cloud-based data mining application, using the IBM Watson IoT Platform and the Data Science Experience (DSX) tool. To analyze the data, Kibana was used to perform advanced analytical tasks. Kibana is an open-source analysis and visualization platform, used to monitor, search, analyze and visualize data through a variety of graphs, for example, charts and tables.

Artificial Intelligence tools were used to validate the sensory data in real time based on the model implemented with Watson Machine Learning.

The devices associated with the applications were tested throughout their development, using the tools provided by the IBM Watson IoT Platform, which provides REST APIs. The models offered by Watson were used for training, evaluation and implementation. We used IBM Watson Studio, offering a high degree of security, both in terms of operation and of persistence over time, and the possibility of recovery. This approach allowed us to offer critical notifications in case of abnormal readings from our sensors regarding the users’ health and safety.

Feedback from the end-users gathered through co-creation sessions is a valuable tool, which facilitated the improvement of the project design. These sessions provided an important insight into the users’ interaction with the system, and researchers received suggestions on how to better satisfy the needs of the older people and their caregivers. Furthermore, the feedback was successfully collected throughout the pilot run through a set of standardized questionnaires for assessing the users’ satisfaction in all pilot sites.

With great support from the Brasov City Council’s Department for Social Services, two groups of users were involved, one for the older people end-users and one for their caregivers, with each of them for their specific application. The aim of this co-creation phase was to identify their specific needs and see how useful it would be for them to use mobile devices and these applications. The same model was applied to both user groups, resulting in 14 older people and seven caregivers providing feedback on their own version of the application. This led to a set of specifications and a set of redesign requirements adapted to users’ needs that were later implemented. All these requirements improved the system, with some referring to the sensors’ communication and data collection, while others referred to some of the application’s features. After discussion sessions with the users, improvements were made for receiving the alerts from the sensors and other notifications and for the application itself, with the user now being able to select, from an improved configuration page, what features/modules they want to use or see.

The experience and results of the NOAH project provide a good basis for further applied research on the definition and implementation of new services for the benefit of older people, adding to issues related to personal safety and issues related to the security of their neighborhood. As a result, another AAL-funded project, “SAfety of elderly people and Vicinity Ensuring—SAVE”, is currently underway.

## 4. System Architecture

The NOAH system was designed with Cloud technologies in mind, either being used as a software ecosystem (SaaS—*Software as a service*) or as infrastructure (IaaS—*Infrastructure as a service*). The main technologies within the NOAH system are Java, Android, MySQL, IoT, MQTT, JavaScript and REST API, all of which are Cloud-related.

**Figure 1:**
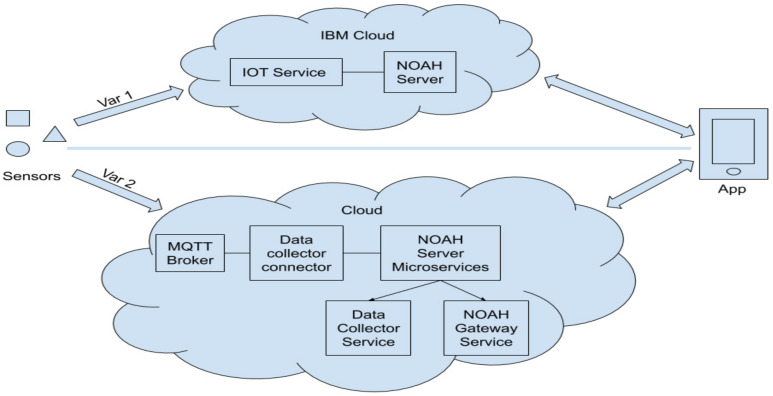
Architecture overview.

There were two iterations of the design. The first (Figure 1—Var 1) had the purpose of developing the features required by the system as fast as possible and obtaining a prototype that could be evaluated with real users, without the need to develop a protocol and environment-related adapters. This variant worked in the SaaS context and made use of readily available tools, boilerplates and software systems in the IBM Cloud (*IoT Service* and *Compose for MySQL*), formerly known as Bluemix. The programming was done in a low-code environment (Node-RED, also offered by the IBM Cloud). The second variant (Figure 1, Var 2), working in the IaaS context, focused on the optimization of costs, scalability and performance. This variant required the development of the final form APIs (*Application Programming Interfaces*) and related software modules in general-purpose programming languages (Java, Python, etc.). The architecture of the latter was based on microservices 12 [33] and allows the deployment of the NOAH Cloud application in a containerization environment (such as Docker).

For both variants, the flow is the same: the sensors send their data through the Internet using the MQTT protocol (*Message Queuing Telemetry Transport*), secured by PKI infrastructure (certificate-based security and hybrid cryptosystems). An MQTT broker (in the prototyping phase, this was included in IBM Cloud’s IoT Service) receives the data and forwards it to the collector software component (a web application that exposes an API), which communicates with the MySQL relational database management system, which retains the data. The user interface connects through dedicated REST APIs (*Representational state transfer APIs*) (the gateway component of the system) to the Cloud application. In the prototyping phase, the collector and the gateway were merged into a Node-RED web application. In the final implementation, they were two separate microservices.

The structure used to exchange data between the gateway and the user interfaces, formalized using the JSON (*Javascript Object Notation*) format, is identical for both variants of the architecture, such that the mobile applications can connect to both variants.

The security of these communication channels is ensured by the Public Key Infrastructure (PKI), the use of SSL/TLS certificates and by a hybrid cryptographic system. Regarding the user interfacing, the secured HTTPS protocol is involved, authentication is based on username–password pairs and authorization uses JSON Web Tokens (JWT).

The gateway component provides four types of information:

- User-related information;
- Alerts: warnings related to technical or non-technical aspects of the devices. Alerts provide details about the status of the sensors, the connection to the system or the battery level and situations in which the sensors are in a certain position, which is unnatural for a person’s routine. Examples include situations in which the front door or the refrigerator door have been open for too long;
- Notifications: warnings related to changes in the behaviour of the monitored person. They are generated by the behavioral analysis module integrated in the system and represent an aspect of the person’s way of life, which must be analyzed and on which it must be intervened with in an appropriate way;
- Statistical data: a log of change in the data taken from the set of sensors, over a certain period, which may be relevant for an analysis performed by specialized personnel.

The microservices-oriented architecture and the chosen protocols and technologies (Spring Boot) allow the deployment of the system on many different types of infrastructures (from a simple server to cloud services, such as IBM Cloud, Amazon’s AWS, Microsoft’s Azure, etc). Even if the prototyping was done on IBM Cloud, the final version of the services could run easily on other infrastructures. The IoT devices make use of the MQTT protocol, so they are not dependent on a particular proprietary technology.

### 4.1. Prototyping Architecture

**Figure 2:**
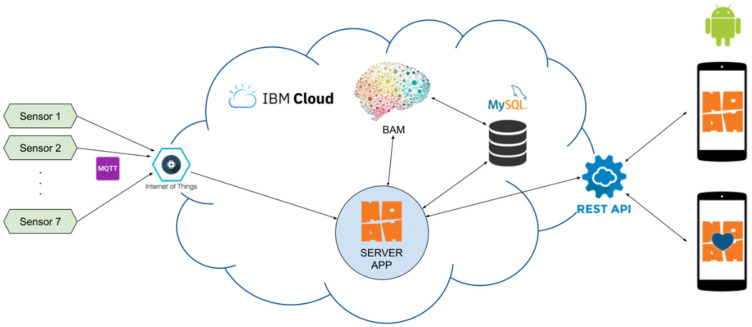
The architecture of the prototype system.

The server side was developed and is hosted by the IBM Cloud, as shown in Figure 2. This requires the continuous running of two main services: *Internet of Things Platform* (IoT) and *Compose for MySQL*.

The IoT platform communicates with the registered equipment and has the role of collecting data from the sensors and transmitting them to the server application. The *Compose for MySQL* service provides support for storing and querying data needed to run the system.

The BAM (*Behavioral Analysis Module*), developed in Python, processes data from sensors to identify behavioral patterns and provides an estimate of the well-being of monitored individuals; the server application reads the BAM outputs and converts them into useful notifications or alerts for users.

The client part of the system is represented by two mobile applications: one for caregiver users (relatives, social workers, friends) and one for end-users (the older people). These applications are developed natively in Android, which can be used on a wide range of smart mobile devices (smartphone or tablet PC).

The communication between the application components, respectively, between the client and the server, is made through the REST type APIs on the HTTPS protocol. Android applications build HTTPS requests corresponding to user intentions and commands and interpret the response, providing information through the user interface.

**Figure 3:**
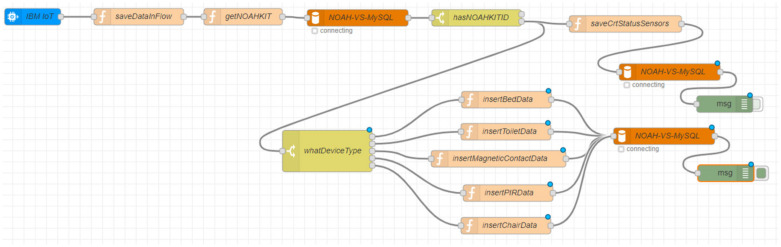
Collector flow in Node-RED.

Figure 3 depicts the flow of the collector implemented in Node-RED. Most of the blocks used to create the flow were readily available and needed minimal configurations. Some JavaScript was required to adapt and enrich the data structures. The collector includes a buffer for the current status of all the sensors, which is very useful for top performance in the mobile applications.

### 4.2. Microservices-Based Architecture

The architecture implemented (see Figure 1) involves a server application built by interconnecting three microservices that fulfil different functionalities. Thus, there is a service that facilitates communication with the connected sensors by incorporating an MQTT broker and which, through a connector, sends the information to the second microservice, which collects this information and stores it. The third microservice is the interface between the server and the client, which exposes the paths for HTTPS requests that serve the information to Android mobile applications.

The server component of the NOAH system is a modular application, with the microservices being developed in different technologies. For instance, the communication part with the connected devices was developed in the Python programming language, and the collection, data processing and customer service were developed in the Java language, using the Spring Boot framework.

This application aims to transfer and process data from the database and serve them in a standardized form in JSON format to Android applications for end-users and caregivers. When a request arises, a path is called to a resource that will process the request and make the necessary transformations to build the response.

## 5. Database

To store the data received from the sensors and other details required for running the NOAH Cloud application, the MySQL relational database management system was chosen.

**Figure 4:**
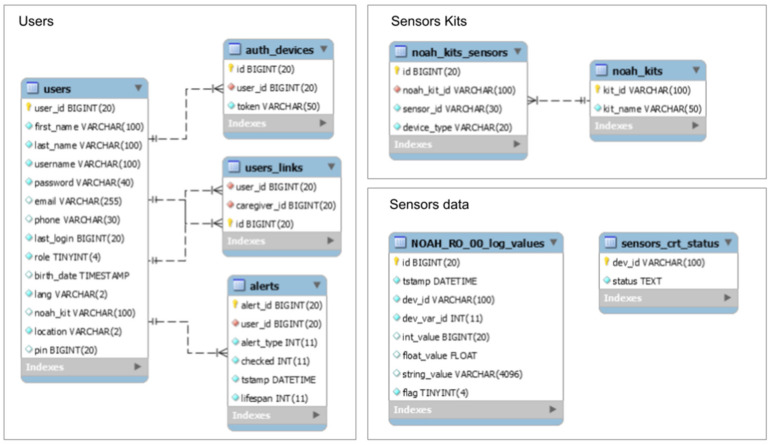
NOAH database.

The sensors are grouped into kits (tables *noah_kits* and *noah_kits_sensors*) and the sensor data are partitioned in different tables by kit; the structure of such a table (*NOAH_RO_00_log_values*) is given in Figure 4. The partitioning helps the access time performance, but it also helps with the users’ data isolation.

The tables store data as follows:

- alerts: stores the alerts generated by the system for users (technical is related to the infrastructure, and notifications are generated, for example, by the BAM module);
- auth_devices: this is used by the auto-login system; when a user logs into the application (either for the caregiver or the end-user), a record with a unique device token is associated with the user; the record is deleted from the table at logout;
- contacts: each end-user can choose two contacts to call directly from the application, in case of emergency; this table stores data about these people;
- noah_kits: stores system-recognized kits (ID and name);
- noah_kits_sensors: contains data about all installed sensors (identifiers and types) and membership in a NOAH kit;
- {noah_kit} _ {number} _log_values (for example, NOAH_RO_00_log_values): stores the data obtained from the sensors associated with the corresponding kit;
- sensors_crt_status: keeps the latest values of the parameters of the sensors connected to the system;
- users: stores details about registered users and their preferences (for example, user interface language); if the user is an end-user, then he has an associated code, which allows caregiver users to associate it in the NOAHCare application, so they can view and receive data about it;
- users_links: contains the associations between caregiver users and end-users.

This solution optimizes the persistence of the collected data. With this partitioning logic, there is a visible link between sensors and their data, and there are no concerns about performance when querying the database to save or retrieve data to be processed. It also provides a faster way to aggregate data in order to build charts for the users.

From the performance perspective, there is also a table that stores the last received values from sensors to provides this information faster to the user, rather than a query for a large amount of data.

It must be highlighted that the collected data have no direct connection to the user’s identity that they belong to in order to ensure the user’s privacy and add a supplementary layer of security.

## 6. Sensors

Each NOAH kit consists of several IoT sensors:

- BED: pressure sensor for detecting when the end-user sleeps; it is placed under the mattress of the end-user’s bed.
- CHAIR: pressure sensor placed under the cushion of the end-user’s favorite chair/couch (that the older person usually sits on during the day, e.g., the one in front of the TV).
- TOILET: a presence sensor placed near the toilet, used to detect toilet usage.
- CONTACT: contact sensor (magnetic) that can be installed on doors or drawers (e.g., the fridge door, if culinary habits need to be monitored, or the entrance door of the home, if safety is concerned).
- PIR: presence sensor (infrared) for detecting the older people’s presence in the living room.

The IoT components were developed especially for this project by WiMonitor SRL, Parma, Italy, for the University of Parma, a partner in the project. The sensing elements were readily available on the market (PIR, pressure sensor, magnetic contact) and were coupled with a WiFi-capable microcontroller development board. The internal details are proprietary to WiMonitor SRL. The estimation cost for installation is 900 EUR for each location in the case of large-scale implementation and a 50 EURO monthly subscription.

The sensors make use of a MQTT library to communicate with the MQTT broker included in the cloud application. The communication is encrypted using a hybrid cryptosystem and PKI’s certificates. The data are sent in batches, at a fixed interval (1 h), to save energy and prolong battery life. However, it is possible to power the sensors using power adapters.

The values provided by the system are of type on–off (1–0) and are tagged with the timestamp of the event; only transitions from on to off and off to on are recorded. The sensors also send the value (charge) of the battery, to be able to generate alerts for replacing them. The different measurements provided by the sensors are identified through the *dev_var_id* field in the data tables (0 for the value of the sensor and 100 for the battery voltage).

The sensors’ primary goal is to monitor the behaviour of the user, not to influence their life. However, by offering access to the data to the caregivers and users (via the visualization feature on the SAVE application), the system could lead to behaviour changes, the creation of healthy habits and a more active and fulfilling life.

## 7. Client Applications

The interaction among the users happens through three applications: Two mobile applications for the end-users and caregivers (NOAH and NOAHCare, respectively) and one web application for administrators (Admin Centre). These applications aim to provide information about sensors’ data and processing outputs as alerts or notifications, as well as managing the sensors within the system.

The main goal of the NOAH system is to gather data from the older people’s environment and process it to information for their caregivers (formal or informal). The system provides statistical information about sensors’ data, notifications in case there were some changes in the end-user’s behaviour, and alerts about the environment (e.g., the door has been left open).

Depending on their role (caregiver, end-user or administrator), users can see the relevant information to their own interest. The end-users choose which caregiver to trust, by furnishing them with a secret PIN that they must input into the NOAHCare app; thus, they decide who is able to see their data.

Access to the system’s features (through UIs) is granted on roles (rights), and the authentication is done using the username–password pair.

### 7.1. NOAH Application

The NOAH application is developed for older people in order to receive a series of useful alerts (e.g., when a fridge door is left open). They have the possibility to send feedback to the system about how they are feeling in a certain moment, to set two contact persons and call them when needed and see general statistics about their sensors.

**Figure 5:**
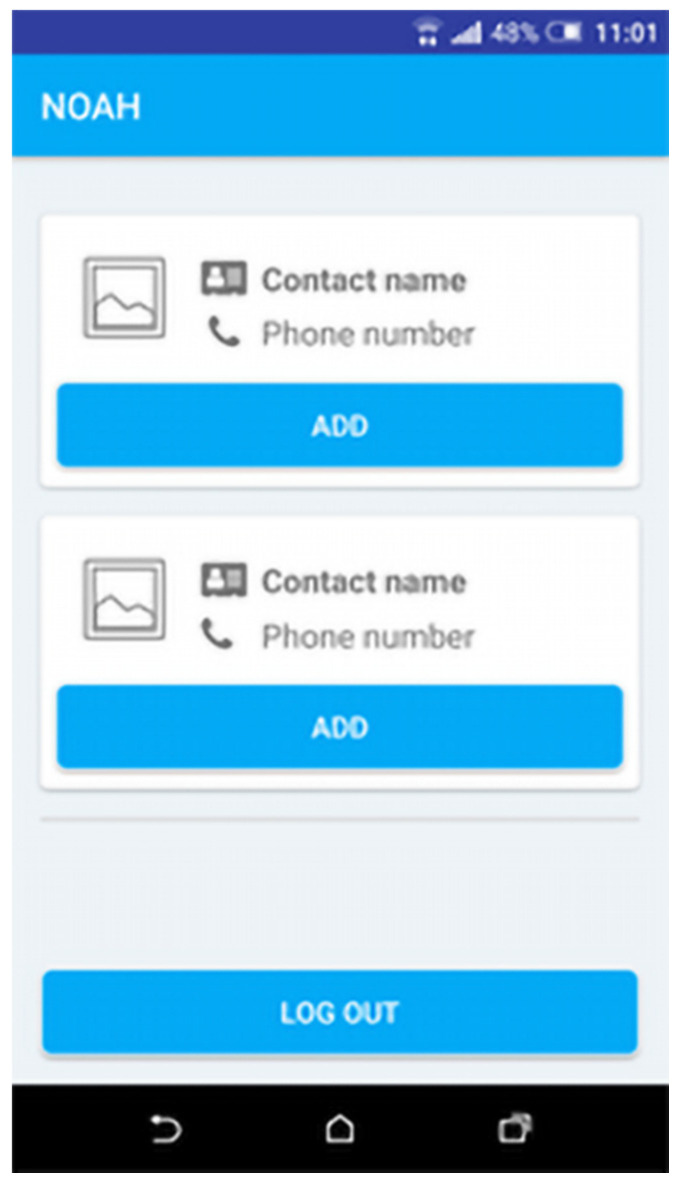
Contacts settings.

**Figure 6:**
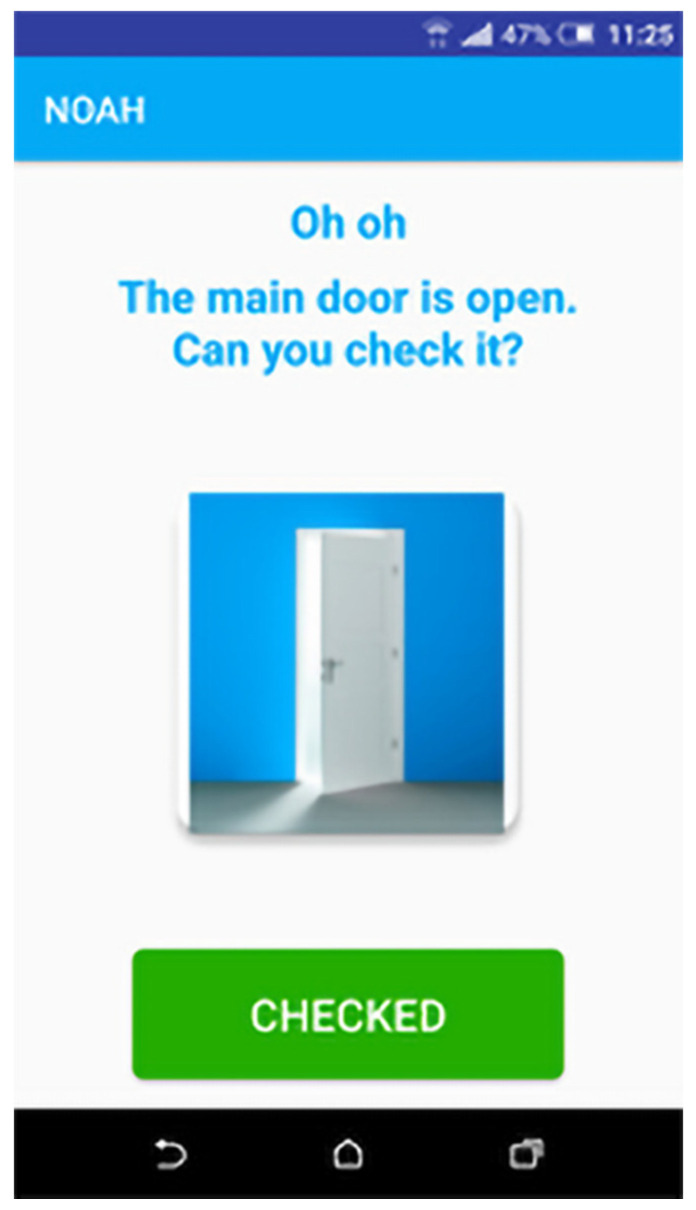
End-user alert.

**Figure 7:**
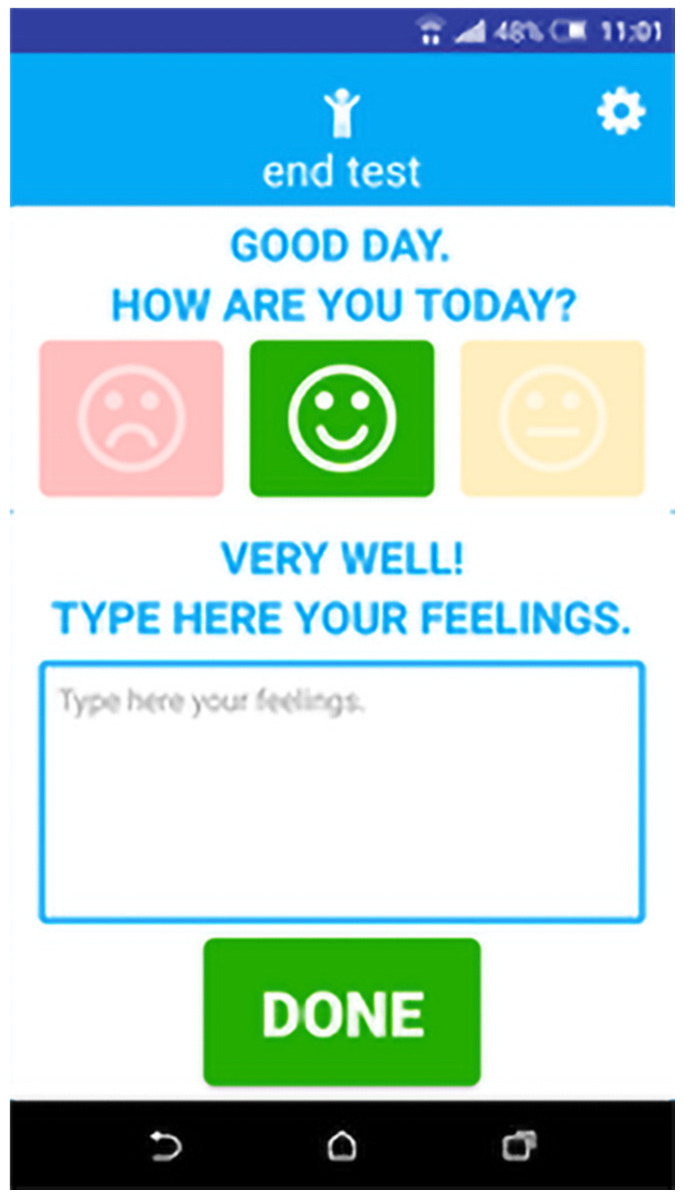
Feedback.

The NOAH application consists of four main features:

- End-user authentication: To authenticate to NOAH app, an end-user must submit the credentials received from a system administrator.
- Contacts: An end-user has the possibility to set two contacts from his/her phone contact list (Figure 5) to be set as quick dial options within the app homepage.
- Alerts: An end-user will receive alerts describing some events happening in his/her environment, such as the entrance door being left open (Figure 6).
- Feedback—An end-user can provide feedback about how he/she is feeling in that moment (Figure 7).

### 7.2. NOAHCare Application

The NOAHCare application is used by the caregivers to keep the older people they take care of under observation. The application offers them access to information, such as the state of the sensors connected in the end-user’s home if there are some changes in the end-user behaviour. They also receive alerts and notifications about sensors, and they can view statistics from different time periods regarding stored data.

The caregiver mobile application usage flow starts with registering an account, logging in and selecting an end-user (from the ones he previously added using the PIN provided by the end-users). At this step, a caregiver can access an application feature to monitor the end-user, such as Sensors’ information (current or statistics over a period), Notifications and Alerts. The notifications are generated by the BAM component and target the end-users’ behaviors; they take the form of advice (e.g., “Today you walked less. You could visit a friend.”). The alerts refer to the state of the sensors and can be related to an action of the end-user (e.g., “You forgot the door open”) or to the functioning of the sensor (e.g., “low battery”, “sensor is disconnected”).

**Figure 8:**
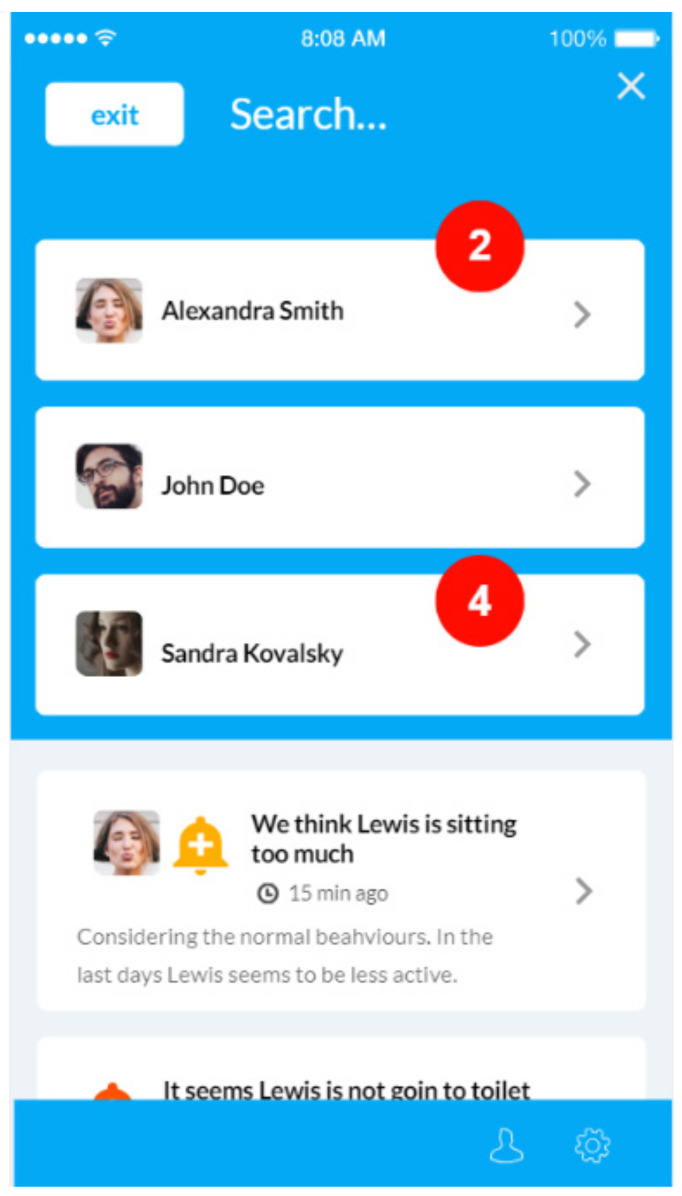
Set end-user.

**Figure 9:**
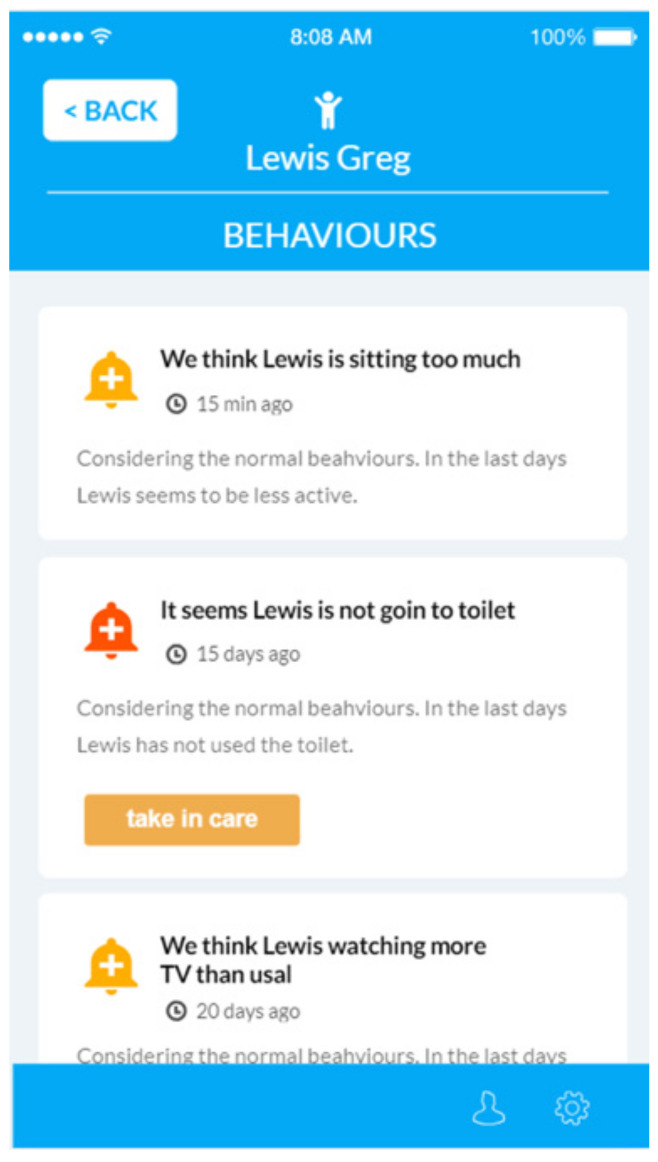
Behaviour.

**Figure 10:**
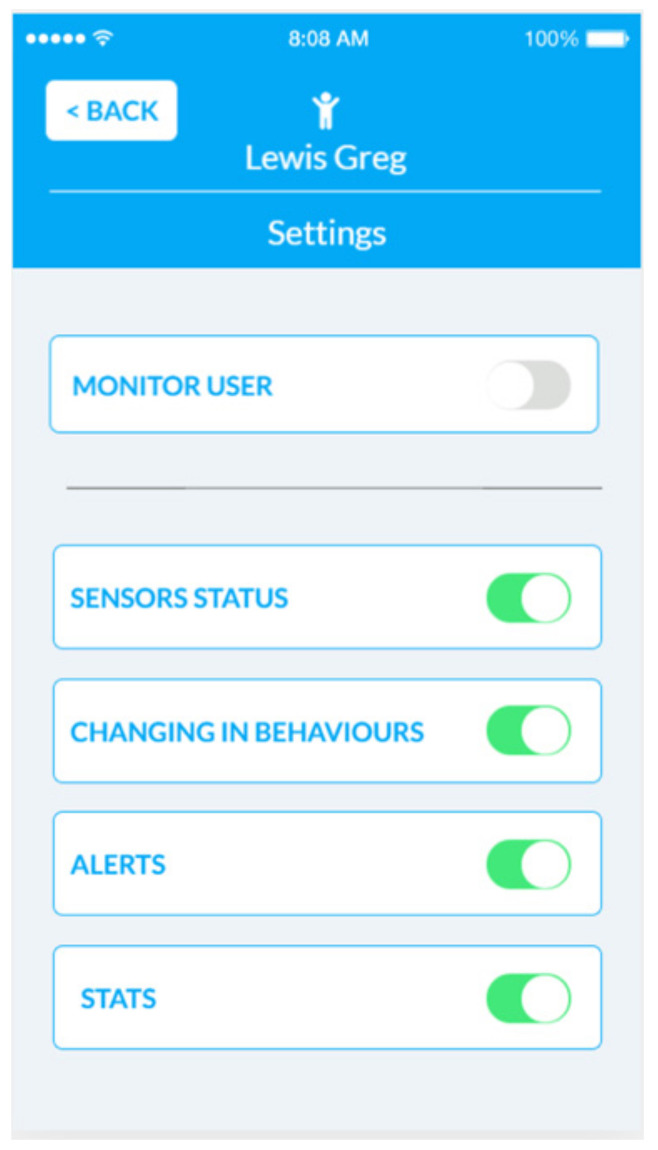
NOAHCare App configuration.

NOAHCare application consists of four main features:

- Caregiver account register and login: A caregiver has the possibility to register a new account by filling their personal information, and after, that they can log into the NOAHCare application.
- End-users management: The flow of a user in this application continues by associating one or more end-users to their account. This step is mandatory for a caregiver user in order to use the application’s facilities. An end-user is linked using a unique PIN number (Figure 8).
- End-user monitoring: The caregiver user can see an overview of data for a specific end-user, the sensor’s status, the changes in their behaviour (notifications) (Figure 9) and alerts.
- Application configuration: A caregiver can configure the application through activating or deactivating features (Figure 10), shown or hidden, respectively, in the app home page.

### 7.3. Admin Centre

The Admin Centre application of the system is a multi-purpose component. It has the role of extracting the information that sensors send, processing it and display it on a friendly interface. The component also offers the possibility of an easy administration of the sensors and kits.

Two types of users, a simple user and an administrator user, can operate it. A simple user can only visualize information, while the administrator user can also insert, edit or delete kits and sensors or the related details.

**Figure 11:**
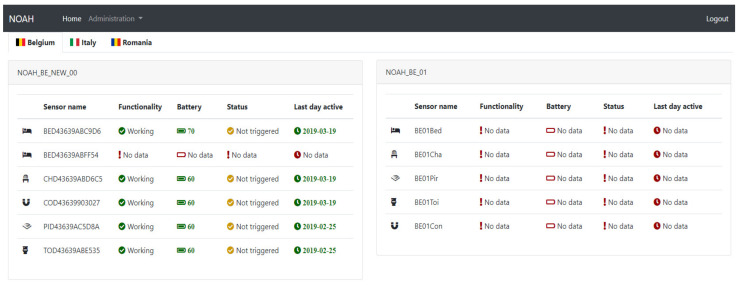
Admin Centre Dashboard.

Once they are logged in, any user will be redirected to a dashboard page containing information about the sensors grouped by kits. The kits are grouped by country for easier filtering (refer to Figure 11).

**Figure 12:**
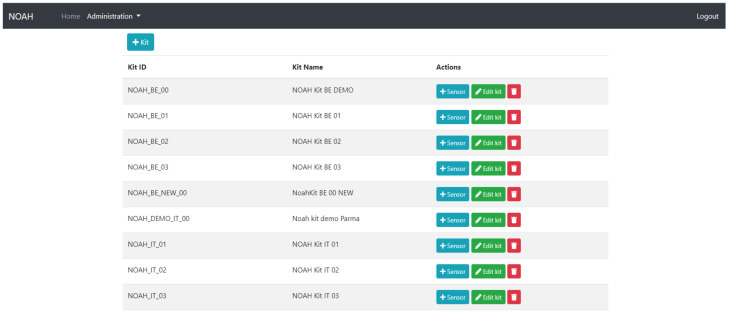
Manage Kits.

**Figure 13:**
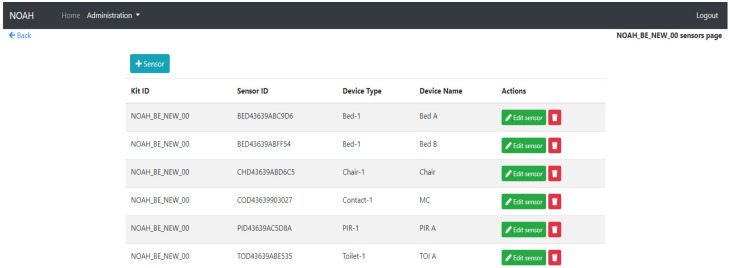
Manage sensors.

An administrator user can manage the sensors and the kits from the interface. It is possible to insert a new kit (Figure 12) or a new sensor (Figure 13), to edit an existing one and to establish a link between these two entities.

## 8. Methods

### 8.1. Participants

The subjects were selected by the Department of Social Services (DSS) within the Brasov City Council, following the NOAH project criteria. The DSS is specialized in working with members of this community. They also supervised the activity of the subjects within the project.

The project activities involving end-users and data collection were made according to the collaboration protocol between DSS and Transilvania University of Brasov. The study and research design were in line with the Declaration of Helsinki.

All subjects were informed of this and signed the written form of consent after having all of their questions answered. All data were rendered completely anonymous. During the NOAH project, a professional caregiver was designated from the DSS for each activity with the end-users.

Originally, 14 Romanian elderly individuals, who participated voluntarily, were included in the present study (2 males and 12 women; Mean age = 72.89 years, Standard Deviation (SD) = 5.08). This group of older people participated in our research study investigating the impact of the IOT system in their own environment, including analyzing behavior in association with sensors’ detection. Only people older than 65 years of age, living alone in their home and not suffering from major chronic diseases or severe disabilities, were included in the study.

### 8.2. Instruments

The participants completed the Adaptive Behaviour Assessment System—Second Edition (ABAS II) [34]. The ABAS II is a questionnaire standardized and validated by an age-stratified Romanian sample [35]. The aim of ABAS II is to assess an individual’s adaptive skills and their ability to live independently. This assessment plays a central role in the evaluation of sensor usage by older people in their homes. It measures functional states of daily skills in areas such as general adaptive composite skills (GAC), conceptual skills (communication, functions, self-direction), social skills (free time, social, practical community usage) and practical skills (including community use, home living, health and safety, self-care) used in the present study. The form used in this study had 239 items and was completed in two sessions. The psychologists from the Department of Social Services carried out the hetero-evaluation for each participant, separately. The inter-rater reliability coefficients on the GAC scores and for the daily skills areas are α = 0.80 s [35]. The test was chosen based on the protocol that is described in the procedure.

### 8.3. Procedure

At the beginning of the procedure, a research team approached the Social Services Department of Brasov City Council and our participants. Participation in the study was exclusively voluntary. Written informed consent from older people and their caregivers was obtained prior to participation in the study. To be included in this study, an individual had to fulfil the following criteria: (a) being more than 65 years old; (b) living alone at home; (c) not suffering from major chronic diseases or severe disabilities; (d) receiving occasional care from relatives or professional caregivers; (e) having a smart phone and internet connection in their home; (f) agreeing to have their home equipped with the home kit and being paired to (at least) one caregiver person in their personal support network. The measurements were collected over a period of 1 year. The caregivers were comprehensively informed about the research project and the conditions of participation. Based on the procedure carried out by Moraru, S.A. et al. [36] and Kristály, D.M. et al. [37], data were collected between October 2019 and September 2020 from sensors installed in the participants’ homes, with an emphasis on the period between mid-February and mid-May 2020, which was the Covid Lockdown period in Romania.

The procedure was as follows: the informed consent process; installation of a new router in the house; installation of sensors (bed, seat, contact, presence and toilet sensor); installation of the telephone application for the older people; presentation of the application; checking the system infrastructure (internet connection, batteries, ergonomic placement in the house, checking the cloud).

### 8.4. Data Analysis

The normal distribution of each variable was tested using the Kolmornov–Smirnov Test. Non-parametric testing, such as the Spearman Coefficient, was used to test the correlation between the bed, chair, contact, presence and toilet sensors associated with the behaviour of the participants. To determine two conditions in which the same people participated in each condition, the Wilcoxon signed-rank test was used. Due to the low number of participants, the observed cases were unusual. The significance level was set at 0.05. The statistical analysis was carried out using SPSS 26.

## 9. Results

### 9.1. Descriptive Results

Information regarding age, sex, educational background (primary school, lyceum, university), family structure (widowed, divorced) and their current health status was collected before starting the measurements. These aspects are recorded in Table 1; the descriptive statistics for all the variables are in Table 2.

### 9.2. Correlation Results

Community use was significantly related to using sensors from the bed (*r*_s_ = −0.76, 95%BCa CI [−0.976–1.00], *p* = 0.27) and social skills were related to using a contact sensor (*r*_s_ = −0.79, 95%BCa CI [−0.044–0.179], *p* = 0.19). There was no other significant association between the variables.

### 9.3. Differences between Periods

To test the differences between the activity recorded by the sensors during the one-year evaluation, we split the data into two six-month periods: October–March 2020 (first analysis) and April–September 2020 (second analysis). Furthermore, we tested the difference between first 6 months and the Covid lockdown period. The results based on the differences between scores using Wilcoxon signed-rank test [38] are presented in Table 3.

## 10. Discussion

As mentioned in the background section, although the use of the IoT system has been shown to have a significant positive impact on maintaining the safety of older people at home, the effect on adaptive behaviour remains unclear. Therefore, the present research investigated the ability of sensors to analyze the behaviour of our participants in their homes during their daily life and in the context of the Covid crisis.

The results showed a low association between daily skills and sensors. Contact sensors were positively associated with social skills, and bed sensors were negatively associated with community use. In previous studies [39,40], the researchers’ reports showed a correlation between using different sensors and behaviour adaptability. Similarly, our results report differences between the activity of sensors during the daily life of the older people in real time [14]. The results also showed differences between first 6 months’ activity and during the 3 months of Covid lockdown, and for the team’s research, it was a positive sign that the data collected were real and that we would receive correct data from our users [4].

In the Background and related work Section, we discussed the relationship between sedentary behaviour and daily activities, but the best protocol for analyzing this process is yet to be determined [41]. In our study, we have reported basic values of continuous activity for the older people group carrying out daily home activities in real time, with each sensor capturing data every hour [5,14]. The results also showed important activity in March and May, a period associated with the lockdown in Romania [17].

Another important discussion is about the efficiency of the sensors while they were functioning. We started collecting data in October 2019, and for the system, it is an important step to understand the needs and gaps between the concerns of our participants and our ability to improve security and the IoT system [15]. To the best of our knowledge, our older people from Social Assistance Services, Brașov, were the first unit in our region to employ the use of bed, chair, contact, presence and toilet sensors, all integrated in one cohesive system.

As a corollary, these answer our research questions:

(1) Do domotic sensors have a long-term relevance and utility? The sensors did detect changes in user behaviour, even one year after installation (October 2019–September 2020).
(2) Did using the system lead to changes in the lifestyle of the older people participating in our pilot? Based on the data collected from the presence sensor, no changes (please see Table 3) were observed during the first and last six months of the study (prior to the Covid restrictions).
(3) Was the users’ behaviour, in their homes during their daily lives, affected by the Covid crisis? The presence sensor was significantly higher during the Covid lockdown period than in the first six months of measurements (Table 3), with a large effect size. We can conclude that during the Covid lockdown, older people were less active and used their personal space (the familiar environment in which the end-user can exercise his autonomy and self-management, i.e., their home) less.

### Limitations

The main limitation of our study is the small number of data points. The aforementioned national lockdown period interfered with the regular functionality check of the sensors, as leaving one’s home was seriously restricted. Our data were collected in real time, but unpredictable system errors could have interfered with the accuracy of the data. Even though we have been in constant contact with the participants, we cannot be sure that they did not keep the sensors in the house incorrectly, which could have potentially led to the interruption of data collection from certain sensors. We cannot discuss prejudices in this study, because contact with the older people was maintained by telephone, and their involvement was based on the feeling that they were not alone, especially during the period of forced isolation.

## 11. Conclusions

In this paper, we combined social evaluation with the technical approach so that the implemented system could acknowledge the users’ feedback. Presenting both aspects together provides more coherence and displays the interconnected nature of our methodology.

Our system focused on a closer interaction between healthcare (caregivers/stakeholders) and technology researchers in order to ensure that this new system addresses the unsolved needs of elders, especially in difficult periods.

In the general context of a system that can exploit an “Internet of Things” approach, NOAH, the powerful system described in this paper, has dedicated home sensors suitable for capturing a vast array of facets from day-to-day living activities in a non-intrusive manner. Sensors connect directly to the Cloud through the home Wi-Fi network, requiring no aggregator node and avoiding the need for dedicated sensor networking.

Commercial Cloud infrastructure was exploited to gather data. The power of the Cloud platform has proven to be the ability to integrate all the sensors (“things”) that can be connected to it. The integrative approach of our system provides all tracked data via a central interface. Data acquired via sensors are transmitted, stored, selected and processed in the Cloud, enabling the user to see it all at a glance. The security of the data in the Cloud is preserved by using on the server-only high-speed encrypted transmissions.

The Agile approach of NOAH resulted in pilot solutions’ co-creation, with role-based user interfaces ranging from simple authentication and prompt/ping to caregiver alerting and administration dashboards. Given the efficiency of the sensors employed and data collection and interpretation since October 2019, this study has proven that this Agile approach was efficient in addressing the needs and gaps between the concerns of our participants and our ability to improve security and the IoT system. To the best of our knowledge, the older people from the Social Assistance Services, Brasov, were the first unit in our region to employ the use of bed, chair, contact, presence and toilet sensors, all combined in one cohesive and fully functional system.

In our study, a significant number of users participated, and we used an industry-standard instrument to conduct, control and validate the experiment on an age-stratified Romanian sample by a procedure that was agreed with our partners in the project. We proved that all the technology used was appropriate, relevant and valued for such a project. Validation of our research was done by confirming that the use of domotic sensors has long-term relevance and utility. We also proved that the sensors detect changes in user behaviour, even one year after installation, by comparing the first 6 months and afterwards, within the Covid lockdown period.

As future work, an evaluation is planned to assess the end-users’ behaviour associated with the use of sensors, by conferring the corresponding significance and by information extraction and discrimination from the context. In addition, more users will be involved to extend the degree of confidence in the results. We also intend to build an overview of our past and present AAL projects, emphasizing the efficiency of the system architecture by adapting the development and operation to modern technologies and by calibrating the offered services to the end-users’ and caregivers’ specific needs, ultimately proving that AAL projects have utility and consistency.

## Figures and Tables

**Table 1:** Descriptive statistics of the participants (older people).

Title 1	Age (Mean; SD)	Sex	Marital Status	Education	Health Issues *
Participants		12 women	78.6% widowed	Primary School 7%	Yes
	72.89; 5.08	2 men	21.4% divorced	Lyceum 71.5%	Yes
				University 21.5%	Yes

* Health issues = all the participants have different age-related chronic illnesses (such as hypertension, heart diseases or diabetes).

**Table 2:** Descriptive statistics * for all the variables included in the study.

Variable	N	Mean	SD	Skewness	Kurtosis
Communication	14	74.07	1.26	−0.944	−0.890
Community use	14	68.57	4.39	−0.962	−0.242
Functional	14	78.79	2.63	−0.817	−0.601
Life/Family	14	68.29	1.06	−1.1	−0.295
Safety/Healthy	14	59.07	1.26	−0.944	−0.890
Leisure time	14	63	6.18	−0.541	−1.10
Self-care	14	74.07	1.14	−0.884	−0.18
Self-Direction	14	72.29	2.86	−0.495	−1.62
Social skills	14	67.5	1.65	−0.597	−1.33
GAC	14	111.07	5.04	0.404	−1.273
Conceptual	14	36.71	2.09	−0.794	0.443
Social	14	24.14	3.67	−0.436	−0.960
Practical	14	46.5	5.17	−1.23	0.684
Bed Sensor	14	27,112.14	16,222.15	0.333	−1.187
Chair Sensor	14	18,935.15	9863.57	0.731	0.255
Contact Sensor	14	13,877.08	5316.97	0.025	−0.916
Presence Sensor	14	39,760.31	24,659.54	−0.082	−1.182
Toilet Sensor	14	10,763.45	6711.73	−0.598	−1.826
Sensors Presence 1–6 months	14	19,780.64	8742.90	−0.771	−0.468
Sensors Presence 7–12 Months	14	16,798.57	9765.60	0.247	−1.476
Sensors Presence Covid Lockdown	14	8920.92	5814.62	0.438	0.597

* Besides the statistical values, the last rows express the number of occurrences.

**Table 3:** Wilcoxon signed-rank test results.

Differences	N	Median	*z*	*p*	*r*—Effect Size
Sensors Presence 1–6 months vs. 7–12 months	14	22,357	−1.35	0.177	-
Sensors Presence 1–6 Months vs. Covid LD	14	10,653	−3.29	0.001	−0.87

N (number of participants); *z* (*z*-scores); *p* (*p*-value, significance level is 0.05); *r* (effect size).

## Data Availability

The raw data supporting the conclusions of this article will be made available by the authors, without undue reservation, to any qualified researcher.
